# Optimizing Genomic Parental Selection for Categorical and Continuous–Categorical Multi-Trait Mixtures

**DOI:** 10.3390/genes15080995

**Published:** 2024-07-29

**Authors:** Bartolo de Jesús Villar-Hernández, Paulino Pérez-Rodríguez, Paolo Vitale, Guillermo Gerard, Osval A. Montesinos-Lopez, Carolina Saint Pierre, José Crossa, Susanne Dreisigacker

**Affiliations:** 1 International Maize and Wheat Improvement Center (CIMMYT), Km 45, Carretera México-Veracruz, Texcoco CP 52640, Estado de México, Mexico; bdjesusvh@gmail.com (B.d.J.V.-H.); p.vitale@cgiar.org (P.V.); g.gerard@cgiar.org (G.G.); c.saintpierre@cgiar.org (C.S.P.); 2 Colegio de Postgraduados, Montecillos CP 56230, Estado de México, Mexico; perpdgo@gmail.com; 3 Facultad de Telemática, Universidad de Colima, Colima 28040, Estado de México, Mexico; osval78t@gmail.com; 4 Louisiana State University, Baton Rouge, LA 70803, USA; 5 Distinguish Scientist Fellowship Program and Department of Statistics and Operations Research, King Saud University, Riyah 11459, Saudi Arabia

**Keywords:** Bayesian decision theory, genomic prediction, continuous traits, categorical traits, genomic parental selection, mixture traits

## Abstract

This study presents a novel approach for the optimization of genomic parental selection in breeding programs involving categorical and continuous–categorical multi-trait mixtures (CMs and CCMMs). Utilizing the Bayesian decision theory (BDT) and latent trait models within a multivariate normal distribution framework, we address the complexities of selecting new parental lines across ordinal and continuous traits for breeding. Our methodology enhances precision and flexibility in genetic selection, validated through extensive simulations. This unified approach presents significant potential for the advancement of genetic improvements in diverse breeding contexts, underscoring the importance of integrating both categorical and continuous traits in genomic selection frameworks.

## 1. Introduction

Since the pioneering study by Meuwissen [1], the use of genomic selection (GS) has experienced consistent growth over the years. Initially, its applications were predominantly observed in the fields of plant [2,3,4,5,6] and animal breeding [7,8,9,10]. However, in more recent times, these applications have transcended into a diverse array of disciplines such as forest preservation and restoration [11,12].

The GS process includes some basic steps: (1) Data collection is performed through genotyping and phenotyping for target traits to establish a training or base population. (2) Model building is accomplished by training a statistical or machine learning algorithm to learn from the data. (3) Once the models have learned, they are applied to individuals in a breeding population for which we have genotypic, but not phenotypic, information. This allows for us to predict breeding values (BVs) for the traits of interest. (4) Finally, the breeder decides which individuals to select to accelerate the genetic improvement of traits over time.

For the second step in the GS process, breeders choose statistical machine learning algorithms based on the type of phenotypic information, which represents the realization of traits. Some traits are continuous, while others are discrete (nominal, ordinal, and counts). Typically, breeding value (BV) predictions are carried out for single traits, although the use of multi-trait predictions has recently become more frequent for continuous traits to exploit correlations between them.

For continuous traits such as plant height, yield, and nutrient content, we can assume a normal distribution for the observed phenotypic data, a notion supported by the familiarity of the normal distributions and available software, such as the popular BGLR package in R-4.2.1 [13], although free distribution approaches such as quantile regression can be performed on skewed traits [14].

For discrete traits, generalized linear models (GLM) are used. In the specific instance of categorical or binary traits, there is an assumption regarding the presence of a latent (unobserved) continuous variable. Such models are commonly referred to as threshold models. The accuracy of predicted BVs is closely linked to the use of suitable statistical machine learning models that match the type of traits. Authors of [15] provide a compelling argument for the use of binary traits instead of continuous traits in genomic prediction models, highlighting the potential benefits in terms of decision metrics such as sensitivity and specificity. Incorporating these viewpoints can enhance the rationale for using appropriate statistical learning algorithms in genomic selection, thereby improving the accuracy and reliability of predictions.

When addressing the challenge of the curse of dimensionality in GS, statistical models are regularized. Regularization techniques play a pivotal role in GS by bolstering model stability, enhancing predictive accuracy, managing high-dimensional data (which arise due to the number of predictors, denoted as p, far exceeding the number of observations, denoted as n), and simplifying the process of selecting relevant genetic markers.

Once an appropriate statistical machine learning model has been trained, the breeder uses it to predict BVs in a candidate set for selection. In the case of single-trait selection, a natural approach is to select individuals with the highest BVs if the trait is continuous. However, if the trait is binary or ordinal, the selection is based on choosing lines with the highest probability of achieving the desired level/category of interest for the breeder.

When selecting for numerous continuous traits, breeders often use selection indices to rank individuals. A selection index generates a single numerical output representing a score for each candidate, reflecting a weighted average of the BVs [16]. The primary challenge associated with using selection indices lies in the intricate calibration of trait weights. In our previous works [17,18], we proposed an alternative approach in which selection is guided by the entire multivariate posterior predictive distribution for each candidate in continuous traits; this methodology is referred to as selection based on the Bayesian decision theory (BDT).

Despite the increasing frequency of continuous multi-trait selection in the existing literature, there is no research addressing how to select candidates when there are two or more ordinal traits. This, regardless of many traits of interest, is measured on ordinal scales. For example, stripe rust resistance is commonly expressed in ordinal scales that reflect the magnitude of symptoms. Similarly, numerous characteristics in animals and plants are represented as either binary or ordinal traits. While some traits exhibit a continuous distribution, they are often measured as ordinal traits for practical reasons. The scenario with multiple ordinal traits is referred to in this work as categorical multi-trait (CM).

A case that is even less explored in the literature, although it is quite common in practice, involves the presence of mixtures of different types of traits. This situation arises when breeders aim to select individuals that excel in one or more continuous traits, as well as in one or more discrete traits simultaneously. Henceforth, we will assume that discrete traits are categorical, implying that the order of categories or levels possesses a natural sequence. We will refer to this scenario as a continuous–categorical multi-trait mixture (CCMM).

To address this lack of investigation, in this paper, we propose a methodology based on the BDT to select the best candidates considering the CM and CCMM scenarios. Our approach is based on the idea that each ordinal trait is associated with an underlying latent trait of continuous nature. By selecting individuals with higher values for the latent trait, we indirectly select the desired category of the trait of interest (if the order goes from lower to higher; otherwise, the order can simply reverse).

By extending this idea to the scenario of T ordinal traits, we have T latent traits that can be assumed to have a multivariate normal distribution. For simplicity, it can be assumed that the latent traits are uncorrelated. This assumption is made because it is not trivial to train statistical machine learning models that contemplate the correlation structure between different ordinal traits in high-dimensional data (n≪p). By assuming uncorrelated latent traits, the complexity and computational cost is reduced significantly, but the price of this assumption might lead to a loss of valuable information. Correlations can capture relationships and trade-offs between traits that could be exploited to make more informed selection decisions. Ignoring these correlations might result in suboptimal selection outcomes. By assuming a multivariate normality of the latent traits, it is possible to calculate the expected a posteriori loss (PEL) using BDT and select those individuals with the lowest PEL values, such as the context of multi-trait selection with continuous traits developed in [18]. 

In the case of CCMM, a practical approach involves modeling continuous traits separately from categorical traits. For continuous traits, a multi-trait linear model can be used to exploit correlations between traits, whereas categorical traits can be modeled assuming they are not correlated. Subsequently, continuous traits and latent traits are assumed to jointly follow a multivariate normal distribution, allowing for the application of BDT as explained in [18].

Both scenarios, CM and CCMM, can be implemented using existing software. Specifically, the posterior predictive distributions of the latent traits and continuous traits can be approximated using the BGLR library [14], while the posterior expected loss (PEL) can be approximated using the MPS library [19].

Hence, the main goal of this research endeavor is to propose a pragmatic methodology for multi-trait selection, targeting multiple ordinal traits (CMs) and CCMMs using GS and applying the BDT. This proposal is primarily directed towards the plant and animal breeding community. To incentivize the acceptance of this methodology, we present the results of a computer simulation study conducted on a long-term breeding program. In this simulation, we considered the CM context, where three ordinal traits, each one with three categories, were simulated. Furthermore, for the case of the CCMM, we simulated one ordinal trait with three categories, along with two continuous traits. Our simulation encompasses two heritability’s, low and moderate, for both the CM and CCMM contexts. In addition, we include a simple real application example considering a CCMM case.

## 2. Materials and Methods

### 2.1. General Structure of Phenotypic and Genomic Data

Suppose our data take the form of $\left\{(x_{i},y_{i}),i=1,\ldots ,n\right\}$ with covariates (molecular markers) $x_{i}={\left(x_{i1},\ldots ,x_{im}\;\right)}^{T}\in \;R^{m},m>1$ and a random response $y_{i}\in R$ in the case of a continuous trait. For a multi-trait continuous response, $y_{i}={\left(y_{i1},\ldots ,y_{it}\right)}^{T}$ is a vector in which each element represents a trait, i.e., $y_{i}\in \;R^{t}$.

In the case of an ordinal trait, $y_{i}$ represents ordered categories, the categories are not equidistant from each other. For example, plant vigor could have three categories (low = 1, medium = 2, high = 3); in this case, *k* = 3. In ordinal multi-trait scenario $y_{i}={\left(y_{i1},\ldots ,y_{it}\right)}^{T}$, each trait can have different categories.

### 2.2. General Model Formulation

In the case of a single continuous trait, the response can be modelled using a linear function of covariates, i.e., $y_{i}=\mu_{0}+\sum_{m=1}^{p}x_{im}\beta_{m}+\epsilon_{i}$, where $\epsilon_{i}∼NIID\left(0,\sigma_{\epsilon }^{2}\right)$, or equivalently, $y_{i}∼NI\left(\mu_{0}+\sum_{m=1}^{p}x_{im}\beta_{m},\sigma_{\epsilon }^{2}\right)$. In multi-trait $y_{i}∼MVN\left(\mu_{i},\Sigma \right)$, where $\mu_{i}={\left(\mu_{i1},\ldots ,\mu_{it}\right)}^{T}$, each element of $\mu_{i}$ is modelled as above, i.e., $\mu_{ij}=\mu_{0j}+\sum_{m=1}^{p}x_{ijm}\beta_{jm}$, for all traits j=1,…,t. Finally, the covariance matrix is denoted as Σ, where the diagonal elements represent the variances of the traits, and the off-diagonal elements represent the covariances between traits

$$\Sigma =\left[\begin{array}{cccc} \sigma_{11} & \sigma_{12} & \ldots & \sigma_{1t}\\ \sigma_{21} & \sigma_{22} & \ldots & \sigma_{2t}\\ \vdots & \vdots & \ddots & \vdots \\ \sigma_{t1} & \sigma_{t2} & \ldots & \sigma_{tt} \end{array}\right].$$

The above formulation for Σ is known as an unstructured covariance matrix, but other configurations exist, such as diagonal, factor analytic and recursive; see details in [20]. Note that we are assuming $y_{i}$ is independent and identically distributed. However, in animal and plant breeding, individuals are often related. This relationship is incorporated by using a kinship and/or pedigree matrix.

In an ordinal trait, the probability of observing a particular category—$P_{r}\left(y=j\right),j=1,2,\ldots ,k$—can be linked to predictors using a non-linear function f(⋅) that in most cases is the probit function [21] that takes the linear predictor $\eta_{i}=\sum_{m=1}^{p}x_{im}\beta_{m}$ as input and a threshold parameter γ∈R associated with an unknown latent variable l∈R. Mathematically, $P_{r}\left(y_{i}=k\right)=\Phi \left(\eta_{i}-\gamma_{k}\right)-\Phi \left(\eta_{i}-\gamma_{k-1}\right),$ where Φ(⋅) represents the cumulative standard normal distribution. The latent variable or latent trait, l, can be interpreted as follows: rather than observing l directly, we observe its categorical version, which is determined by $y_{i}=k$ if, and only if, $\gamma_{k-1}\leq l_{i}\leq \gamma_{k}$.

In turn, $l_{i}=\sum_{m=1}^{p}x_{im}\beta_{m}+\epsilon_{i}$, and it is assumed that $\epsilon_{i}∼N(0,\sigma_{l}^{2})$ with the restriction that $\sigma_{l}^{2}=1$ for the identifiability of the rest of model parameters. It should be noted that intercepts are not included in liability formulation given that threshold parameters act as intercepts, with the restriction that $-\infty <\gamma_{0}<\gamma_{1}<\cdots <\gamma_{k}<\infty$.

### 2.3. Categorical Multi-Trait (CM)

The presence of multiple ordinal traits, here referred to as categorical multi-trait (CM), occurs when breeders are interested in more than one ordinal trait. Consider, for instance, two such traits: “drought tolerance” with categories low = 1, medium = 2, and high = 3 and “fruit quality” with categories poor = 1, fair = 2, good = 3, very good = 4, and excellent = 5. It is noteworthy that the number of levels for each trait may differ. The breeder could be interested in individuals exhibiting high drought resistance and excellent fruit quality.

In this example, it becomes apparent that presuming a multivariate normal distribution would not be prudent, given the discrete nature of the traits. Therefore, a simple choice is to use categorical regression models for each trait independently. For each categorical regression there is a latent variable l∈R. The combination of multiple categorical regressions forms a vector of latent variables $l_{i}={\left(l_{1},\ldots ,l_{t}\right)}^{T},\;l_{i}∼MVN(\eta_{i},\Sigma_{l})$, with $\eta_{i}={\left(\eta_{1},\ldots ,\eta_{t}\right)}^{T}$ and $\Sigma_{l}=Diag(1,\ldots ,1)$.

### 2.4. Continuous–Categorical Multi-Trait Mixtures (CCMM)

Suppose we have t traits; a subset is continuous ($y_{i}$), and the rest are ordinals ($l_{i}$). They jointly form a vector, $y_{i}^{*}={\left(y_{i},l_{i}\right)}^{T}$. By construction, continuous traits are multivariate normal, and for categorical traits, the corresponding latent traits are also multivariate normal. Thus, all the mixed continuous–categorical traits are multivariate normal that can be formulated as $y_{i}^{*}∼MVN(\mu_{i}^{*},\Sigma^{*})$, where $\mu_{i}^{*}={\left(\mu_{i},\eta_{i}\right)}^{T}$ and the variance–covariance matrix $\Sigma *=\left[\begin{array}{cc} \Sigma & 0\\ 0 & \Sigma_{l} \end{array}\right]$.

#### 2.4.1. Posterior Predictive Distribution and Posterior Expected Loss

The posterior distribution for a vector of parameters $\theta \in \Theta ,\;p(\theta |X,Y^{*})$ is obtained using Bayes’ theorem, X is the matrix of molecular markers and $Y^{*}=\left(y_{1}^{*T},\ldots ,y_{n}^{*T}\right)$, where each $y_{i}^{*T}$ was defined above. In GS, $p\left(\theta |X,Y^{*}\right)$ is approximated by the Markov chain Monte Carlo (MCMC) integration technique. The posterior predictive distribution of a candidate line for selection in the context of CCMM scenario is given by $p\left(y_{c}^{*}|x_{c},Y^{*},X\right)=\int_{\theta \in \Theta }p\left(y_{c}^{*}|\theta ,x_{c}\right)p\left(\theta |Y^{*},X\right)\partial \theta$.

By combining the Bayesian decision theory for genomic selection [18], we can compute the posterior expected loss for each candidate:

(1) $${\bar{L}}_{c}=\int_{y_{c}^{*}\in Y_{c}^{*}}\int_{\theta \in \Theta }L\left(F_{y_{c}^{*}}\;,\theta \right)p\left(y_{c}^{*}|\theta ,x_{c}\right)p\left(\theta |Y^{*},X\right)\partial \theta dy_{c}^{*}$$

where $L\left(F_{y_{c}^{*}}\;,\theta \right)$ represents a generic loss function that depends on the multivariate distribution ($F_{y_{c}^{*}}$) of $y_{i}^{*}={\left(y_{i},l_{i}\right)}^{T}$. $L\left(\cdot ,\cdot \right)$ could be any of the loss functions proposed in [17,18]. After computing the posterior expected loss for each candidate, a decision maker could rank each candidate, from minimum to maximum posterior expected loss, and select a fraction of candidates with the lowest posterior expected loss.

Note that in the above formulation, for model identifiability, we supposed that latent traits have a variance equal to one, and they are independent of each other; therefore, $\Sigma_{l}=Diag\left(1,\ldots ,1\right)$. These assumptions are obviously unrealistic; most categorical traits might be least weakly correlated. Additionally, we suppose that every continuous trait is independent of each ordinal trait; therefore, solutions based on the above formulation are suboptimal. To date, breeders do not have any practical approaches to capture the dependence between ordinal traits in genomic selection, let alone in CCMM; consequently, our approach suggests that there is a practical first approach to conduct selection in CCMM cases.

The above proposal can be implemented using existing software. Specifically, BGLR can be used to conduct multi-trait and ordinal regressions separately. The MCMC chains from BGLR can then be used to approximate the posterior expected loss, as given by Equation (1) for each candidate line, using the MPS-0.1.0 R Package [19].

#### 2.4.2. Simulation Study

We simulated a recurring selection plan with ten selection cycles. In each selection cycle, an offspring of full siblings was derived from parents randomly chosen from the entire population. From each offspring, lines of double haploids were randomly generated, resulting in a total of 2000 lines in each cycle. To represent historical evolution and induce linkage disequilibrium, 200 generations of random mating were simulated in a population of 2000 lines segregating for all loci. The allelic frequency was fixed at 0.5. The simulated genetic component follows Mendelian segregation laws for diploid species. The genome was composed of 8000 sites segregating independently of each other.

In the case of CM, three correlated categorical traits were genetically simulated based on three quantitative traits, assuming a full pleiotropic model [22]. The same was carried out for the CCMM design, although in this case, we simulated two quantitative traits and one categorical trait (categorized from a quantitative trait), the three of which were genetically correlated. In both cases, this was carried out by randomly sampling gene effects for all segregating sites from a multivariate normal distribution with a mean of zero and a previously stated variance–covariance, to ensure a genetic correlation of quantitative traits at the first generation of −0.37 between trait 1 and trait 2; a genetic correlation of 0.34 between traits 2 and 3; and a genetic correlation of −0.02 between trait 1 and trait 3. To mimic complex and simple quantitative traits, narrow-sense heritability of 0.3 and 0.6 were assumed for all traits as in [18]. Hereinafter, we will always refer to the traits in terms of narrow-sense heritability ($h^{2}$), given that in the simulation plan we simulated a purely additive model and did not include dominance effects. Each quantitative trait was transformed into an ordinal trait, each one with three categories in the CM scenario. In the case of CCMM, two quantitative traits were treated as continuous, and the third was discretized into three categories.

The population proportion of each category for each categorical trait at the F0 for CM scenario was as follows: 49% for trait 1 category 1 (T1C1), 34% for T1C2, and 17% for T1C3; 49% for T2C1, 23% for T2C2, and 28% for T2C3; and 14% for T3C1, 36% for T3C2, and 50% for T3C3. In the case of CCMM, the proportion of the categorical trait at F0 was 49% (C1), 19% (C2), and 32% (C3). Subsequently, 70% of these lines were used to train the regression model using the BGLR-1.1.2 software.

Thirty percent of the remaining lines were used as a pool of candidate individuals for selection, subjected to a 30% selection pressure. Selection was performed by ranking individuals based on their PEL from lowest to highest. The computation of multivariate posterior predictive distribution for latent traits and PEL were approximated using the MPS R Package, assuming preference for the third category of each trait in CM condition. In the context of CCMM, we assumed the need to increase the genetic values for the two quantitative traits and to increase the frequency of the third category for the ordinal trait. Subsequently, the selected lines were crossed by random mating to form the new improved population. In each selection cycle, the heritability of quantitative traits, the population mean of quantitative traits, and the population proportion in ordinal traits were monitored, among other things. This process was repeated twenty times (Monte Carlo replicates).

#### 2.4.3. Experimental Data

This example illustrates the application of CCMM in wheat data. For this purpose, phenotypic and genotypic information of 300 lines is known. The phenotypic records correspond to five traits, three of which are continuous (GY-B5IR, GY-B2IR, and GY-BLHT) and two are discrete (SR-NJ and YR-NJ). The continuous traits include grain yield (GY) measured in three different selection environments at the CIMMYT experimental field in Ciudad de Obregón, Mexico: optimal environment (B5IR), intermediate drought (B2IR), and late heat stress (BLHT). The discrete traits represent the percentage severity of stem rust (SR) and yellow rust (YR) observed in Njoro, Kenya (NJ). SR and YR traits were placed into four categories according to the sample quartiles. Category 1 represents individuals who experienced the highest severity of the disease (upper quartile), whereas category 4 represents individuals who experienced the lowest severity of the disease (lower quartile). Thus, this categorization implies a preference for the selection of lines with a higher probability of belonging to category 4 (the most resistant). Finally, the genotypic information pertains to single-nucleotide polymorphisms (SNPs) obtained through genotype-by-sequencing (GBS) technology. Raw data are allocated in https://github.com/bjesusvh/PaperGenes2024 (accessed on 1 June 2024). To replicate a realistic scenario that breeders might encounter, we randomly divided the data into a training set (300 lines) and a candidate set (50 lines). The training data were used to calibrate the statistical model. Subsequently, predictions were made for the candidate lines, with each line ranked based on the posterior expected loss using Kullback–Leibler loss. Technical details are provided in the Results Section.

## 3. Results

### 3.1. Simulated Data

Table 1 provides a summary of the main findings from the simulation study across two heritability scenarios ($h^{2}=0.3$ and $h^{2}=0.6$) and under the CM and CCMM frameworks. The “goal” column refers to the selection objectives. In the CM framework, the objective was to decrease the population proportion of trait category 1 (less desired) across all traits over time, while increasing the proportion of trait category 3 (more desired). For category 2, the best-case scenario expected a decrease in frequency relative to category 3, although an increase was not entirely negative for improvement purposes, as category 2 is more desirable than category 1. According to the results, most scenarios achieved the selection objective. When comparing the percentage of genetic gain at the end of the selection program relative to the first cycle, large percentages corresponded to cases where there was a statistically significant difference (α=0.01) as a function of time. In the CCMM scenario, both continuous traits were anticipated to increase their genetic value, and for the discrete trait, category 3 was the most desired and an increase of frequency expected. Based on the results, the expected objective was achieved for the the two continuous traits and the discrete trait, with greater percentage genetic gains when $h^{2}=0.6$.

**Figure 1:**
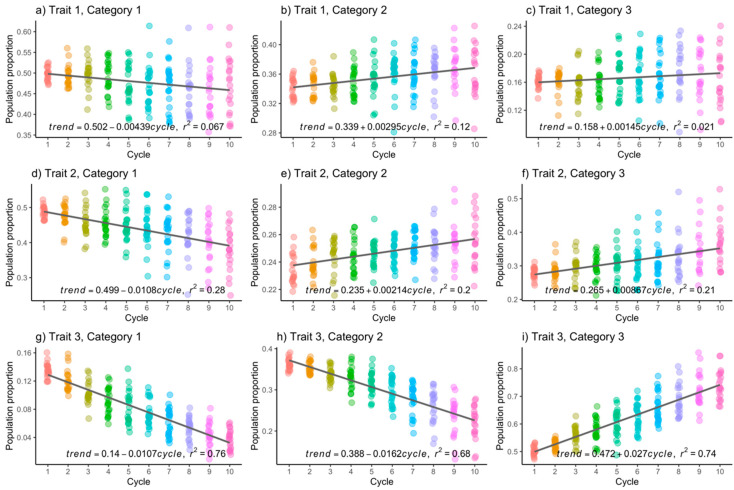
Population frequencies of each category in each trait were examined when heritability was set at 0.3. The *x*-axes represent the selection cycles, whereas the *y*-axes represent the population proportion. Each dot represents a result from a Monte Carlo replicate.

**Figure 2:**
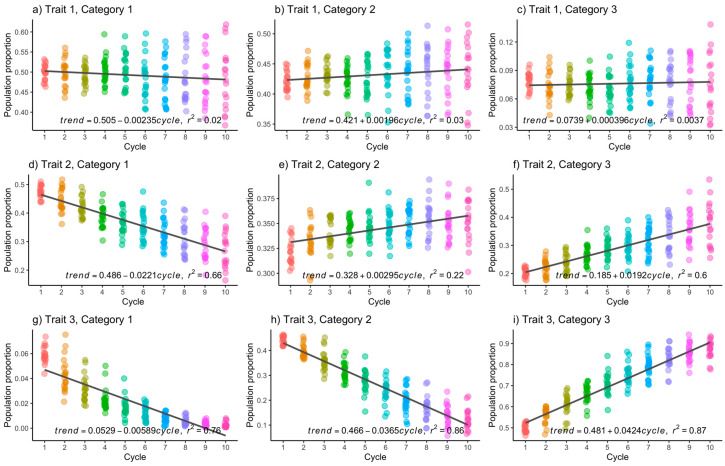
Population frequencies of each category in each trait were examined when heritability was set at 0.6. The *x*-axes represent the selection cycles, whereas the *y*-axes represent the population proportion. Each dot represents a result from a Monte Carlo replicate.

Figure 1 and Figure 2 depict the population frequencies of each category in every categorical trait. Each point on the graphs represents the population proportion in a Monte Carlo replication, with the *x*-axis representing selection cycles. Particularly, Figure 1 presents the results when $h^{2}=0.3$. The trend of population proportions in category 3 of the three traits (Figure 1c,f,i) is observed to show an increase of frequency across selection cycles: 0.15% (T1C3), 0.86% (T2C3), and 2.70% (T3C3) per selection cycle. These increases follow a linear trend, although there is a non-uniform variance in the proportions over time, prompting a formal comparison in the following section using a non-parametric test.

While the primary focus is on the third category of each trait, which is the desired one to increase, it is also important to analyze the trend in the other categories. Initially, there is a negative trend in the first categories of the three traits, as expected. Furthermore, for category 2, there is a slight increase per selection cycle in trait 1 (0.29%) and trait 2 (0.21%), while in trait 3, there is a negative trend (1.62%). When $h^{2}=0.6$, the trend is similar. Specifically, for the third category, the increases per selection cycle were 0.03% (T1C3), 1.92% (T2C3), and 4.24% (T3C3), as shown in Figure 2c, 2f, and 2i, respectively. The increases for the second and third traits are noted to be greater when $h^{2}=0.6$, in comparison to when $h^{2}=0.3$, but this is not the case for trait 1.

**Figure 3:**
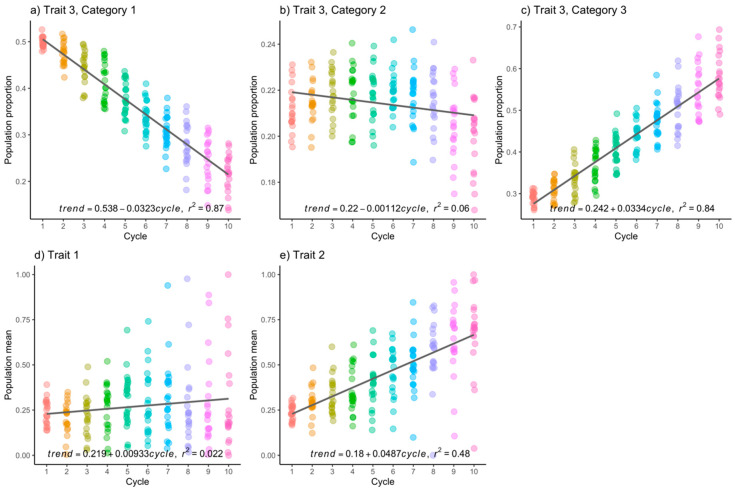
(**a**–**c**) Population frequencies of each category in the categorical trait, and (**d**,**e**) the population mean of the continuous trait were examined when heritability was set at 0.3. The *x*-axes represent the selection cycles, whereas the *y*-axes represent the population proportion, or the population mean. Each dot represents a result from a Monte Carlo replicate.

For the CCMM scenario and $h^{2}=0.3$, both continuous traits show the tendency to increase over time, as desired. However, for trait 1 (Figure 3d), this increase was only 0.93% per cycle, while for the second continuous trait, the increase per cycle was 4.87% (Figure 3e). For the third category of the third trait, the increase per cycle was 3.33% (Figure 3c). In conclusion, there were genetic gains in almost all traits.

**Figure 4:**
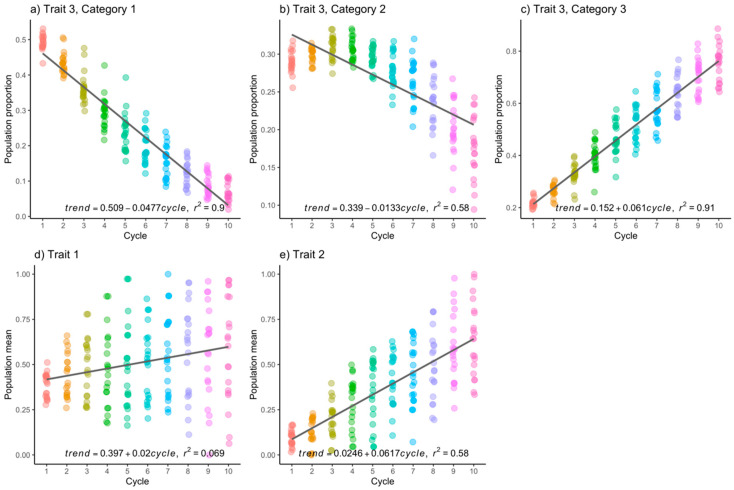
(**a**–**c**) Population frequencies of each category in the categorical trait, and (**d**,**e**) the population mean of the continuous trait were examined when heritability was set at 0.6. The *x*-axes represent the selection cycles, whereas the *y*-axes represent the population proportion, or the population mean. Each dot represents a result from a Monte Carlo replicate.

In the scenario of $h^{2}=0.6$ in CCMM, the average genetic gain per cycle, assuming a linear trend, was 2% for trait 1 (continuous) (Figure 4d), 6.1% for trait 2 (continuous) (Figure 4e), and 6.1% in category 3 of trait 3 (Figure 4c). In summary, the selection goal was achieved in two out of the three traits.

Upon examination of Figure 1, Figure 2, Figure 3 and Figure 4, it becomes apparent that the observed data exhibit deviations from the linear trend. Additionally, there is a discernible trend of increasing variance with the progression of improvement cycles. In response, we conducted the non-parametric Kruskal–Wallis test to assess whether there are statistically significant differences in population means across time, and the non-parametric Mann–Whitney U test with Bonferroni correction was used to conduct multiple means comparisons.

The outcomes of the Kruskal–Wallis test reveal significant disparities (α=0.05) in population means for the following combinations within the CM scenario with $h^{2}=0.3$: T1C1, T2C1, T3C1, T1C2, T2C2, T3C2, T2C3, and T3C3. Similarly, in the CM scenario with $h^{2}=0.6$, significant differences are observed for T2C1, T3C1, T2C2, T3C2, T2C3, and T3C3. Table 2 and Table 3 present the results of the Mann–Whitney U test with Bonferroni correction for multiple mean comparisons (α=0.05) for T2C3 and T3C3 under $h^{2}=0.3$ and $h^{2}=0.6$, respectively. In these tables, “S” denotes statistically significant differences in mean comparisons between cycles, and “NS” indicates non-significant differences.

Note that in the case of T3C3 ($h^{2}=0.3$), significant differences relative to cycle 1 are observed from cycles 9 and 10 onwards, as shown in Table 2. These discrepancies became apparent towards the latter stages of the breeding program. Conversely, for T3C3 (*h*^2^ = 0.6), a statistical significance in differences was established as early as the initial selection cycle.

For T2C3 (*h*^2^ = 0.6) and T3C3 (*h*^2^ = 0.6), statistically significant differences relative to cycle 1 began to appear as early as selection cycle 3, as depicted in Table 3. Furthermore, nearly all conceivable mean comparisons yielded statistically significant results.

Furthermore, differences were also observed when testing CCMM. In the case of trait 2 (continuous, $h^{2}=0.3$), disparities relative to cycle 1 began to emerge as early as cycle 4, with the majority of possible comparisons yielding statistically significant results, as presented in Table 4. For the categorical trait, significant differences were observed across all three categories. However, Table 4 presents result solely for category 3 of the trait (the preferred category), revealing that significant differences were apparent from early selection cycles, with nearly all possible comparisons being statistically significant. Regarding trait 1 (continuous), no differences were observed, indicating that this trait remained neutral with no genetic gain or loss.

Similarly, when $h^{2}=0.6$, more pronounced significant differences were observed. For trait 2 (continuous), genetic gains from cycle to cycle of selection were higher compared to when $h^{2}=0.3$. This conclusion is drawn from the comparison of the slopes of the trend lines in both cases (Figure 3e vs. Figure 4e) and is further confirmed by the non-parametric test, where a greater number of statistically different comparisons were found (Table 5). Additionally, the categorical trait also displayed significant differences across all three categories of the trait, and unlike when $h^{2}=0.3$, these differences were of a greater magnitude. Comparisons for category 3 (the preferred category) are presented in Table 5. Once again, trait 1 (continuous) showed neither genetic gain nor loss.

### 3.2. Experimental Data

**Figure 5:**
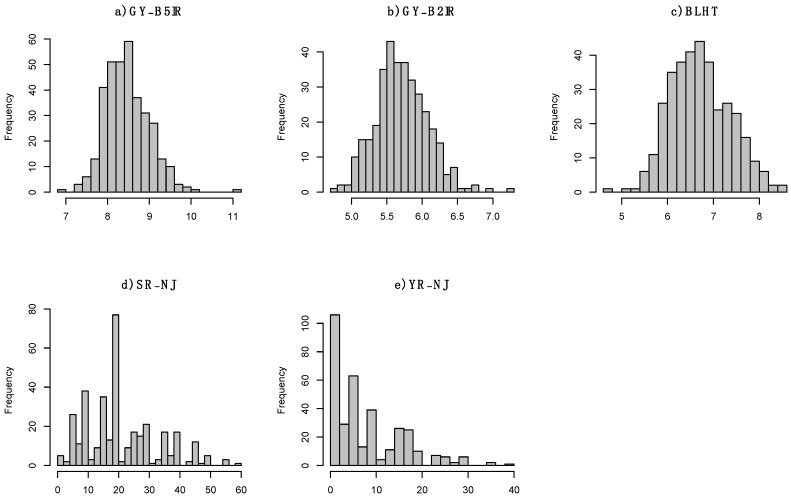
Histograms of the five traits. Plots (**a**–**c**) show approximately symmetrical normal distributions for the continuous traits. Plots (**d**,**e**) exhibit highly skewed distributions for the discrete traits.

Figure 5 depicts the distribution of the traits involved in the selection. The continuous traits (Figure 5a–c) exhibit symmetrical distributions. The Shapiro–Wilk test indicates that GY-B2IR and BLHT are normally distributed (α=0.01). Therefore, we pragmatically assumed that all three traits follow a multivariate normal distribution. However, the discrete traits are asymmetrical, and normality is questionable. Therefore, we categorized into four categories as described previously.

In addition, our example supposes that there is genomic information for 50 candidates for selection. In Appendix A, R codes and detailed explanations are provided to replicate the exercise; with minor modifications, users can adapt the code to suit their own needs.

**Figure 6:**
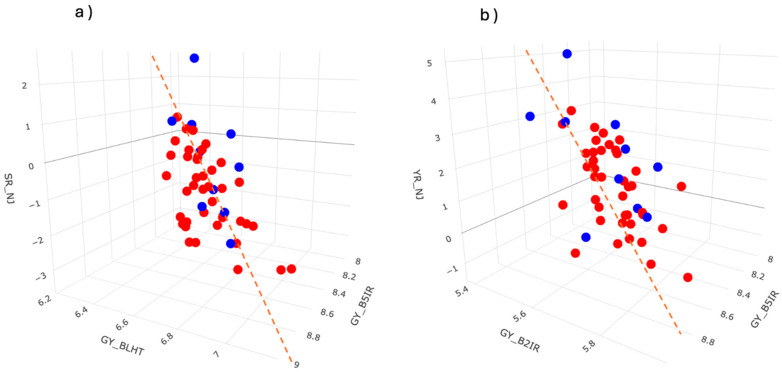
Three-dimensional plots of GEBVs for each trait under selection. Blue dots represent selected individuals, while red dots represent non-selected lines. (**a**) Traits GY_BLHT, GY_B5IR and SR_NJ; (**b**) Traits GY_B2IR, B5IR and YR_NJ.

Therefore, each one the 50 candidates lines have a rank based on PEL. Suppose we are interested in identifying the top 10 candidates. The pair plots in Figure 6 illustrate the estimated GEBVs for each trait (including latent BVs for ordinal traits), highlighting (blue dots) the lines that should be selected. The red dotted lines indicate the regions where we expect the selected lines to fall, given the need to increase BVs across all traits. Notably, almost all the selected lines fall within these regions, confirming that these lines are the best according to the PEL criterion.

**Figure 7:**
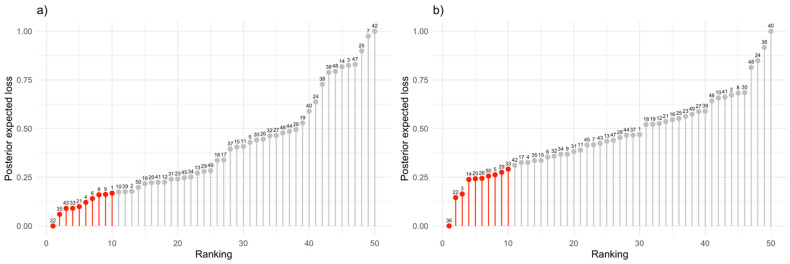
Ranking vs. posterior expected loss for each of the 50 candidates. The label above each bar represents the line ID number. (**a**) Results considering all traits (continuous and categorical); (**b**) results considering only continuous traits and ignoring categorical traits.

Now, suppose we ignore the presence of ordinal traits and perform selection considering only the three continuous traits. The question that arises is whether the ranking of each line remains the same compared to when selection considers all traits. Figure 7 shows the discrepancies when selecting based on both approaches. By ignoring the ordinal traits, the top 10 lines identified are 36, 22, 3, 14, 20, 26, 50, 5, 29, and 33, whereas when considering all traits, the top 10 lines are 22, 35, 43, 33, 21, 4, 6, 8, 9, and 1. Note that only lines 22 and 33 appear in both cases, although they have different rankings. Observing the ranking of all lines, it is evident that the order is entirely different in both scenarios. These types of discrepancies result in suboptimal selection in breeding programs, highlighting the importance of selection in the context of CCMM.

Remember that this exercise mimics a real CCMM scenario. In practice, in the absence of statistical methodologies to address this challenge, selection is generally performed considering only continuous traits, or using categorical traits as continuous. But, this simple example illustrates the importance of using appropriate methodologies in an easy way to implement.

Observing the ranking of all lines (Figure 7a,b), it is evident that the order is entirely different in both scenarios. Such discrepancies result in suboptimal selection in breeding programs, underscoring the importance of selection in the context of CCMM. These discrepancies highlight the critical role of comprehensive selection methods that integrate both continuous and discrete traits. By neglecting discrete traits, valuable genetic variations that could enhance the overall performance and adaptability of the breeding lines are overlooked. Furthermore, the imbalance created by focusing solely on continuous traits can lead to an overemphasis on certain characteristics while neglecting others that are equally important for the success of the breeding program. This selective pressure might inadvertently favor traits that are advantageous under specific conditions but detrimental in broader contexts, thereby reducing the robustness and versatility of the selected lines.

The exclusion of discrete traits not only limits the genetic diversity but also reduces the potential for innovation in breeding strategies. Discrete traits often represent key qualitative attributes, such as disease resistance or drought tolerance, which are critical for developing resilient and high-performing crop varieties. Ignoring these traits can make the breeding outcomes less applicable to real-world agricultural challenges, where such attributes play a crucial role in ensuring sustainable production and food security.

## 4. Discussion

This study introduces a novel methodology for optimizing genomic parental selection in breeding programs by integrating both categorical and continuous traits using Bayesian decision theory (BDT) and latent trait models within a multivariate normal distribution framework. The approach enhances selection precision and flexibility, capturing the genetic architecture of diverse traits more accurately. Extensive simulations and a real-world application demonstrate its practical utility and potential to advance genetic improvements across various breeding contexts.

The methodology significantly improves selection precision by incorporating both trait types, addressing the challenge of dimensionality, and ensuring computational efficiency and practical implementation using existing software. This comprehensive approach allows for breeders to achieve more informed selection decisions, particularly for traits with categorical or ordinal distributions, such as disease resistance or quality traits. The successful application in simulations with various trait combinations and heritability’s underscores its robustness and practical value. The simulation results, summarized in Table 1, highlight the differential outcomes under various heritability scenarios (*h*^2^ = 0.3 and *h*^2^ = 0.6) and selection frameworks (CM and CCMM). Notably, the CCMM framework yielded significant genetic gains for continuous traits and achieved selection objectives for categorical traits, albeit with some variability. This suggests that CCMM models can effectively balance the trade-offs between improving continuous traits and achieving categorical trait targets, which is critical for comprehensive breeding programs.

Despite its strengths, our study acknowledges limitations, such as the assumption of uncorrelated latent traits, which may lead to the loss of valuable trait correlation information. Future research should focus on incorporating correlations between latent traits and expanding validation across different species and breeding programs to ensure the methodology’s robustness and generalizability. Future research could explore the application of this methodology to real-world breeding programs, evaluating its effectiveness in practical scenarios. Integrating this approach with other genomic selection tools could further enhance its adoption and effectiveness in breeding programs.

## 5. Conclusions

This study introduces a novel methodology to optimize genomic parental selection in scenarios involving CM and CCMM. By leveraging the BDT, we effectively address the complexities of selecting candidates across both ordinal and continuous traits. Our approach underscores the importance of considering both trait types simultaneously, enabling precise and flexible genetic selection.

Specifically, we observed significant genetic gains in almost all traits, with a notable increase in the continuous traits by 4.87% per cycle and the categorical trait by 3.33% per cycle under the CCMM framework when heritability was set at 0.3. Furthermore, for a heritability of 0.6, the genetic gains were 2% and 6.1% per cycle for the two continuous traits, respectively, and 6.1% per cycle for the categorical trait. These results highlight the method’s effectiveness in achieving the selection objectives and demonstrate the practical utility of our approach. Results from our experimental data further support the efficacy of the proposed method, showing a consistent improvement in trait selection accuracy and overall breeding efficiency. This reinforces the practical applicability of our methodology in real-world breeding programs.

The integration of latent trait models within a multivariate normal distribution framework ensures comprehensive and efficient selection, validated through extensive simulations. This unified approach for CM and CCMM scenarios represents a significant advancement in genomic selection. Future research should refine these models and explore their broader applications, promising substantial genetic improvements in various breeding programs.

## Figures and Tables

**Table 1:** Summary of the results obtained from the simulation study of a breeding program under two scenarios, categorical multi-trait (CM) and continuous–categorical multi-trait mixture (CCMM), and two heritability conditions.

CM: Categorical Multi-Trait					$h^{2}=0.3$		$h^{2}=0.6$	
Trait	Type	Category	Notation	Goal	Achieved	Average % Change	Achieved	Average % Change
1	Categorical	1	T1C1	Decrease	Yes	−5.36	Yes	−2.95
1	Categorical	2	T1C2	Increase/decrease	Yes	6.65	No	4.39
*1*	*Categorical*	*3*	*T1C3*	*Increase*	*Yes*	*2.47*	*Yes*	*−4.72*
2	Categorical	1	T2C1	Decrease	Yes	−21.93	Yes	−42.62
2	Categorical	2	T2C2	Increase/decrease	Yes	8.72	Yes	9.09
*2*	*Categorical*	*3*	*T2C3*	*Increase*	*Yes*	*31.06*	*Yes*	*84.84*
3	Categorical	1	T3C1	Decrease	Yes	−71.97	Yes	−95.29
3	Categorical	2	T3C2	Increase/decrease	Yes	−38.96	Yes	−71.58
*3*	*Categorical*	*3*	*T3C3*	*Increase*	*Yes*	*47.47*	*Yes*	*74.45*
CCMM: Continuous–Categorical Multi-trait Mixture					$h^{2}=0.3$		$h^{2}=0.6$	
**Trait**	Type	Category	Notation	Goal	Achieved	Average % change	Achieved	Average % change
1	Continuous	-	T1	Increase	Yes	23.96	Yes	50.77
2	Continuous	-	T2	Increase	Yes	185.26	Yes	572.10
3	Categorical	1	T3C1	Decrease	Yes	−56.32	Yes	−85.88
3	Categorical	2	T3C2	Increase/Decrease	Yes	−5.19	Yes	−39.03
3	Categorical	3	T3C3	Increase	Yes	100.61	Yes	246.36

**Table 2:** Results of the Kruskal–Wallis test and Mann–Whitney U test for results of trait 2 category 3 (T2C3) and trait 3 category 3 (T3C3) for $h^{2}=0.3$.

Trait 2 Category 3, $h^{2}$ = 0.3									
*p*-Value = 2.14 × 10^−6^ from the Kruskal–Wallis Test									
*p*-Values from the Mann–Whitney U Test Using the Bonferroni Correction									
	Cycle 1	Cycle 2	Cycle 3	Cycle 4	Cycle 5	Cycle 6	Cycle 7	Cycle 8	Cycle 9
Cycle 2	NS								
Cycle 3	NS	NS							
Cycle 4	NS	NS	NS						
Cycle 5	NS	NS	NS	NS					
Cycle 6	NS	NS	NS	NS	NS				
Cycle 7	NS	NS	NS	NS	NS	NS			
Cycle 8	NS	NS	NS	NS	NS	NS	NS		
Cycle 9	S	S	NS	NS	NS	NS	NS	NS	
Cycle 10	S	S	NS	S	NS	NS	NS	NS	NS
Trait 3 Category 3, $h^{2}$ = 0.3									
*p*-value = 7.55×10^−29^ from the Kruskal–Wallis test									
*p*-values from the Mann–Whitney U test using the Bonferroni correction									
	Cycle 1	Cycle 2	Cycle 3	Cycle 4	Cycle 5	Cycle 6	Cycle 7	Cycle 8	Cycle 9
Cycle 2	S								
Cycle 3	S	S							
Cycle 4	S	S	NS						
Cycle 5	S	S	NS	NS					
Cycle 6	S	S	S	NS	NS				
Cycle 7	S	S	S	S	NS	NS			
Cycle 8	S	S	S	S	S	NS	NS		
Cycle 9	S	S	S	S	S	S	NS	NS	
Cycle 10	S	S	S	S	S	S	S	NS	NS

**Table 3:** Results of the Kruskal–Wallis test and the Mann–Whitney U test for results of trait 2 category 3 (T2C3) and trait 3 category 3 (T3C3) for $h^{2}=0.6$.

Trait 2 Category 3, $h^{2}$ = 0.6									
*p*-Value = 3.90 × 10^−23^ from the Kruskal–Wallis Test									
*p*-Values from the Mann–Whitney U Test Using the Bonferroni Correction									
	Cycle 1	Cycle 2	Cycle 3	Cycle 4	Cycle 5	Cycle 6	Cycle 7	Cycle 8	Cycle 9
Cycle 2	NS								
Cycle 3	S	NS							
Cycle 4	S	S	NS						
Cycle 5	S	S	S	NS					
Cycle 6	S	S	S	NS	NS				
Cycle 7	S	S	S	NS	NS	NS			
Cycle 8	S	S	S	S	NS	NS	NS		
Cycle 9	S	S	S	S	S	S	NS	NS	
Cycle 10	S	S	S	S	S	NS	NS	NS	NS
Trait 3 Category 3, $h^{2}$ = 0.6									
*p*-value = 2.74 × 10^−32^ from the Kruskal–Wallis test									
*p*-values from the Mann–Whitney U test using the Bonferroni correction									
	Cycle 1	Cycle 2	Cycle 3	Cycle 4	Cycle 5	Cycle 6	Cycle 7	Cycle 8	Cycle 9
Cycle 2	S								
Cycle 3	S	S							
Cycle 4	S	S	NS						
Cycle 5	S	S	S	NS					
Cycle 6	S	S	S	S	NS				
Cycle 7	S	S	S	S	S	NS			
Cycle 8	S	S	S	S	S	S	NS		
Cycle 9	S	S	S	S	S	S	S	NS	
Cycle 10	S	S	S	S	S	S	S	NS	NS

**Table 4:** Results of the Kruskal–Wallis test and the Mann–Whitney U test for results of continuous–categorical multi-trait mixture simulations for $h^{2}=0.3$.

Trait 2 Continuous, $h^{2}$ = 0.3									
*p*-Value = 8.46 × 10^−22^ from the Kruskal–Wallis Test									
***p*** **-Values from the Mann–Whitney U Test Using the Bonferroni Correction**									
	**Cycle 1**	**Cycle 2**	**Cycle 3**	**Cycle 4**	**Cycle 5**	**Cycle 6**	**Cycle 7**	**Cycle 8**	**Cycle 9**
Cycle 2	NS								
Cycle 3	NS	NS							
Cycle 4	S	NS	NS						
Cycle 5	S	NS	NS	NS					
Cycle 6	S	S	NS	NS	NS				
Cycle 7	S	S	S	NS	NS	NS			
Cycle 8	S	S	S	S	S	NS	NS		
Cycle 9	S	S	S	S	S	S	NS	NS	
Cycle 10	S	S	S	S	S	S	NS	NS	NS
Cycle 10	NS	NS	S	NS	S	S	S	NS	NS
Trait 3 Category 3, $h^{2}$ = 0.3									
*p*-value = 1.79 × 10^−34^ from the Kruskal–Wallis test									
*p*-values from the Mann–Whitney U test using the Bonferroni correction									
	Cycle 1	Cycle 2	Cycle 3	Cycle 4	Cycle 5	Cycle 6	Cycle 7	Cycle 8	Cycle 9
Cycle 2	NS								
Cycle 3	S	NS							
Cycle 4	S	S	NS						
Cycle 5	S	S	S	NS					
Cycle 6	S	S	S	S	NS				
Cycle 7	S	S	S	S	S	NS			
Cycle 8	S	S	S	S	S	S	NS		
Cycle 9	S	S	S	S	S	S	S	NS	
Cycle 10	S	S	S	S	S	S	S	S	NS

**Table 5:** Results of the Kruskal–Wallis test and the Mann–Whitney U test for results of continuous–categorical multi-trait mixtures simulations for $h^{2}=0.6$.

Trait 2 Continuous, $h^{2}$ = 0.6									
*p*-Value = 8.46 × 10^−22^ from the Kruskal–Wallis Test									
***p*** **-Values from the Mann–Whitney U Test Using the Bonferroni Correction**									
	**Cycle 1**	**Cycle 2**	**Cycle 3**	**Cycle 4**	**Cycle 5**	**Cycle 6**	**Cycle 7**	**Cycle 8**	**Cycle 9**
Cycle 2	NS								
Cycle 3	S	NS							
Cycle 4	S	NS	NS						
Cycle 5	S	S	NS	NS					
Cycle 6	S	S	S	NS	NS				
Cycle 7	S	S	S	NS	NS	NS			
Cycle 8	S	S	S	S	NS	NS	NS		
Cycle 9	S	S	S	S	S	NS	NS	NS	
Cycle 10	S	S	S	S	S	S	NS	NS	NS
*p*-value = 1.79 × 10^−34^ from the Kruskal–Wallis test									
*p*-values from the Mann–Whitney U test using the Bonferroni correction									
	Cycle 1	Cycle 2	Cycle 3	Cycle 4	Cycle 5	Cycle 6	Cycle 7	Cycle 8	Cycle 9
Cycle 2	S								
Cycle 3	S	S							
Cycle 4	S	S	S						
Cycle 5	S	S	S	S					
Cycle 6	S	S	S	S	NS				
Cycle 7	S	S	S	S	S	NS			
Cycle 8	S	S	S	S	S	S	NS		
Cycle 9	S	S	S	S	S	S	S	NS	
Cycle 10	S	S	S	S	S	S	S	S	NS

## Data Availability

Raw data are allocated in https://github.com/bjesusvh/PaperGenes2024.

## Appendix A

**“Optimizing Genomic Parental Selection for Categorical and Continuous–Categorical Multi-trait Mixtures”**

The following lines of R codes illustrate how to apply the BDT approach in a CCMM context. **Chunk #1** loads the required packages and data. Three hundred lines are used to train the statistical learning models, and 50 lines are used as candidates for selection, emulating a real scenario in genomic selection. We used Bayesian ridge regression (BRR) to impose quadratic penalization to regression coefficients. BGLR is instructed to save the MCMC chains of the regression coefficients using the argument saveEffects = TRUE. After iterating 50,000 times, the first 20,000 MCMC chains are discarded as burn-in, and thinning at lag five. The remaining MCMC chains are used for inference in this example.

**Chunk #2** fits the regression models saving MCMC chains in the “out” folder that exists in our working directory. The Multitrait function of BGLR [20] is used to fit the multivariate regression model on the three continuous traits. Setting saveEffects = TRUE allows the MCMC chains of the covariance matrix to be saved. Subsequently, categorical regression models are independently fitted to the remaining two discrete traits.

**Chunk #3** starts reading MCMC samples to create the array of regression coefficients and covariance matrix $\Sigma^{*}$. This matrix should have 15 distinct entries corresponding to the variances and covariances among the five traits. Recall that this matrix has the following structure: $\Sigma^{*}=\left[\begin{array}{cc} \Sigma_{3\times 3} & 0_{3\times 2}\\ 0_{2\times 3} & \Sigma_{l_{2\times 2}} \end{array}\right].$ We detail each component of this matrix below:

$\Sigma_{3\times 3}$ is the covariance matrix corresponding to quantitative traits,

$$\Sigma_{3\times 3}=\left[\begin{array}{ccc} \sigma_{1}^{2} & \sigma_{12} & \sigma_{13}\\ \sigma_{12} & \sigma_{2}^{2} & \sigma_{23}\\ \sigma_{13} & \sigma_{23} & \sigma_{3}^{2} \end{array}\right].$$

${\Sigma_{l}}_{2\times 2}$ represent the covariance matrix (diagonal by model identifiability of regression coefficients in ordinal regression) of latent traits.

$${\Sigma_{l}}_{2\times 2}=\left[\begin{array}{cc} 1 & 0\\ 0 & 1 \end{array}\right]$$

$0_{3\times 2}$ is the diagonal of $\Sigma^{*}$.

$$0_{3\times 2}=\left[\begin{array}{cc} 0 & 0\\ 0 & 0\\ 0 & 0 \end{array}\right]$$

Therefore, $\Sigma^{*}$ is conformed as follows:

$$\Sigma^{*}=\left[\begin{array}{ccccc} \sigma_{1}^{2} & \sigma_{12} & \sigma_{13} & 0 & 0\\ \sigma_{12} & \sigma_{2}^{2} & \sigma_{23} & 0 & 0\\ \sigma_{13} & \sigma_{23} & \sigma_{3}^{2} & 0 & 0\\ 0 & 0 & 0 & 1 & 0\\ 0 & 0 & 0 & 0 & 1 \end{array}\right]$$

This matrix is filled to use in MPS R Package as the following: provide the upper triangular matrix including the main diagonal in the order: $\sigma_{1}^{2},\sigma_{12},\sigma_{13},0,0,\sigma_{2}^{2},\sigma_{23},0,0,\sigma_{3}^{2}$, 0, 0, 1, 0, 1.

The last line in **chunk #3**, the PEL, is approximated using the MPS R Package. In addition to the PEL, the ‘out’ object includes the posterior punctual BVs of each candidate for each trait and the ranking of each candidate.
